# The Effect of a New Therapy for Children with Tics Targeting Underlying Cognitive, Behavioral, and Physiological Processes

**DOI:** 10.3389/fpsyt.2016.00135

**Published:** 2016-08-11

**Authors:** Julie B. Leclerc, Kieron P. O’Connor, Gabrielle J.-Nolin, Philippe Valois, Marc E. Lavoie

**Affiliations:** ^1^Centre d’études troubles obsessionnels-compulsifs et tics, Centre de recherche de l’Institut universitaire en santé mentale de Montréal, Montreal, QC, Canada; ^2^Laboratoire d’étude des troubles de l’ordre de la psychopathologie en enfance, Département de psychologie, Université du Québec à Montréal, Montreal, QC, Canada; ^3^Département de Psychiatrie, Université de Montréal, Montreal, QC, Canada; ^4^Laboratoire de Psychophysiologie Cognitive et Sociale, Montreal, QC, Canada

**Keywords:** Tourette disorder, tics, children, treatment, cognitive–behavioral therapy, psychophysiological

## Abstract

Tourette disorder (TD) is characterized by motor and vocal tics, and children with TD tend to present a lower quality of life than neurotypical children. This study applied a manualized treatment for childhood tics disorder, *Facotik*, to a consecutive case series of children aged 8–12 years. The *Facotik* therapy was adapted from the adult cognitive and psychophysiological program validated on a range of subtypes of tics. This approach aims to modify the cognitive–behavioral and physiological processes against which the tic occurs, rather than only addressing the tic behavior. The *Facotik* therapy lasted 12–14 weeks. Each week 90-min session contained 20 min of parental training. The therapy for children followed 10 stages including: awareness training; improving motor control; modifying style of planning; cognitive and behavioral restructuring; and relapse prevention. Thirteen children were recruited as consecutive referrals from the general population, and seven cases completed therapy and posttreatment measures. Overall results showed a significant decrease in symptom severity as measured by the YGTSS and the TSGS. However, there was a discrepancy between parent and child rating, with some children perceiving an increase in tics, possibly due to improvement of awareness along therapy. They were also individual changes on adaptive aspects of behavior as measured with the BASC-2, and there was variability among children. All children maintained or improved self-esteem posttreatment. The results confirm the conclusion of a previous pilot study, which contributed to the adaptation of the adult therapy. In summary, the *Facotik* therapy reduced tics in children. These results underline that addressing processes underlying tics may complement approaches that target tics specifically.

## Introduction

### Definition and Symptoms

Tourette disorder (TD) is considered a motor disorder in the neurodevelopmental disorders section of the DSM-5 (1). TD is diagnosed by multiple motor tics and at least one vocal tic present for at least 1 year. This child-onset disorder appears to be a complex condition with the changing nature of tics evolving over time in frequency, intensity, localization, and complexity (2). Children and adolescents are the most affected by TD with a prevalence rate between 0.3 and 0.9% (3).

Studies report that children with TD are more likely to experience daily struggles in several spheres of activities (4). Cutler et al. (5) showed that 66% of 57 young participants reported some physical consequences associated with tics (e.g., pain, aches, physical discomfort). In a school setting, tics may interfere with academic performance and produce difficulty concentrating, writing, or reading (6). Children with TD may also experience relationship problems because they can be victimized when their tics are severe and complex, and they can be stigmatized or have more conflicts with their parents and teachers than other children (7–9). Hoekstra and colleagues (10) reported an increase in emotional problems over time in TD children and a higher rate of cognitive difficulties than children in the general population (*p* < 0.05) (11). Consequently, children with TD tend to present a lower quality of life than neurotypical children (5, 12, 13).

About 85% of individuals with TD report at least one comorbid disorder (14, 15). The most frequent comorbidity in children with TD is attention deficit hyperactivity disorder and oppositional defiant disorder (16, 17), but they can also show obsessive–compulsive disorder, anxiety disorder, and depressive disorder (18, 19). The severity of the comorbidity seems to worsen the quality of life of these children often more than tics. The variety of symptoms then interferes in daily functioning, leading to several impairments in children with TD and comorbidity (20, 21).

### Behavioral Therapies

Canadian, American, and European clinical guidelines recommend medication plus a cognitive–behavioral treatment for reducing tics (22–24). Behavioral therapies are recommended as evidence-based interventions to manage tics, and behavioral approaches have taken several forms depending on whether the tic is conceptualized.

The comprehensive behavioral intervention for tics (CBIT) proposed by Woods and colleagues (25) is mainly based on the habit reversal treatment [HRT, Ref. (26)], which was reported to be effective for both children and adults (27–31). In addition to HRT components, such as awareness training, relaxation, competing response, contingency management, and generalization training, CBIT emphasizes the importance of addressing environmental factors that can influence tic manifestations. This 8-week treatment also uses strategies such as psychoeducation about tics and function-based interventions. CBIT appears to be effective for tic reduction in children and adults with TD (32–34). However, the premonitory urge remained unchanged across therapy (35), whereas, in theory, it should decrease with the negative reinforcement process. Therefore, the mechanisms underlying tics and therapeutic processes remain unclear.

In treatment by exposure and response prevention [ERP, Ref. (36)], the aim is to reduce tics by breaking the negative reinforcement loop between the premonitory urge and the tic itself. The individual learns to tolerate the premonitory urge and resists the appearance of tics for longer and longer periods (response prevention). A study comparing two treatment protocols ERP/HR in 43 participants with TD (aged 7 to 55 years old) showed no significant difference between groups in reduction of tics, where 58% of the participant in the ERP group and 28% of the participant in the HR group showed a decrease of at least 30% on the YGTSS (37). However, some children are unable to feel and detect the premonitory urges (38), and the therapy to resist the tic may be sometimes emotionally difficult for the child because of the pressure to succeed.

### Cognitive Psychophysiological Treatment

An elaboration of the functional role of tics in sensorimotor regulation is found in O’Connor’s (39) cognitive psychophysiological model. This model conceives of tics as serving a function of sensorimotor autoregulation, while decreasing tension in muscles inappropriately contracted. Tension in TD seems characterized by a cycle where the muscle is inappropriately prepared prior to execution (40). For example, during an activity, the individual with a tic is preparing too quickly for an immediate response, but, at the same time, preparing more muscles with more effort than necessary. This preparation is inappropriate so the tic action relieves, in part, through local tension release. Electromyographic (EMG) recordings of tic-affected muscles show that these muscles are rarely associated with zero tension and have a greater difficulty compared to non-affected muscles achieving different degrees of tension rather just an all or nothing state of tension [(41) and replication is in preparation]. People suffering from tics also subjectively report chronic tension, and Hoogduin et al. (36) reported high overall muscle tension as a consistent feeling in all patients, when identifying premonitory urges. The originality of this approach is its targeting of excessive overall sensorimotor activations by addressing cognitions, behaviors, and physiological strategies, which engender excessive tension leading to and maintaining tics, rather than learning a competitive response to the tic or to the urge to tic.

The cognitive psychophysiological [CoPs; Ref. (40)] treatment for tics was developed in order to focus on the processes influencing thoughts and behaviors underlying tics, rather than working exclusively on the tic *per se*. Several cognitive factors are targeted in the CoPs treatment, such as anticipation, rigid beliefs (e.g., about action and organization), a judgmental style of thinking, attentional focus, and a perfectionistic style of planning action involving over-activity and over-preparation. This thinking can encourage the tendency to complete several tasks rapidly and at the same time (a style termed over-activity), together with an over-investment in preparation for action by recruiting redundant muscles and employing more effort than necessary (a style termed over-preparation). People with tics frequently experienced rigid thinking about how they should act and appear, resulting in inflexible black and white thoughts, which impair adaptation (42). In addition, meta-cognitive factors, as defined by O’Connor as thoughts about performing the tic and expectations or beliefs about tic onset, are targeted along with how people with tics evaluate and judge situations at high risk for eliciting tics (42). These cognitive factors also interact with physiological factors such as an increased sensorimotor activation, leading to hypersensitivity and over-reactivity and so, as a circular linking, to tic onset (42). A behavioral target of this therapy is to break the negative reinforcement cycle between the tic onset and the immediate relief of the accumulation of muscular tensions caused by the heightened sensorimotor activation (40). There is evidence of tension building up, prior to ticking, and subjective reports of relief, post-ticking (40). The aim of the CoPs treatment is to help the individual in understanding how these cognitive–behavioral and physiological factors lead to tension and how gradually addressing and modifying them can prevent tension build-up and tic onset, while increasing self-control.

An open trial showed the efficacy of CoPs treatment in adults with tics compared to waitlist with a 6:1 ratio (43). Results showed that 10 of the 85 participants completely reduced tic onset after therapy (gains maintained at 6-month follow-up). Prior results also showed efficacy in tic reduction in adults with or without medication, following CoPs treatment (44, 45). The therapy was applied to five adolescents with TD, in a pilot study (46). Results showed a decrease in tic frequency and intensity and improvement in social functioning for the five participants. The CoPs treatment has also been adapted for children with TD addressing explosive outbursts (EO). Results showed a decrease of EO frequency of at least 34% for four participants out of six. Another participant showed a 75% decrease in posttreatment, but did not complete the follow-up assessment. The last participant showed a 67% decrease between the beginning of therapy and follow-up, despite an increase of EO frequency at baseline assessment (47). Finally, a single-case design study of the CoPs treatment addressing tic severity in childhood was conducted with 11 children aged 8–12 years old (48). A decrease of 29.8% of tic onset was observed posttreatment (*p* < 0.001, *d* = 0.97), and the decrease was monitored over 1 year. Results showed a decrease of at least 1 SD in measures, post 12 months.

After this pilot study, a manualized version of the treatment protocol in children termed *Facotik* has been finalized (49), and the aim of the current study is to evaluate its efficacy in a larger consecutive case series. Based on previous research, a decrease of tic severity was expected after treatment. The efficacy of the treatment adapted for children will have important implications for the intervention in TD and whether addressing the underlying sensorimotor processes is sufficient to reduce tics.

## Materials and Methods

### Participants

The recruitment was carried out through the *Centre d’études troubles obsessionnels-compulsifs et tics – Institut universitaire en santé mentale de Montréal*. Consecutive referrals were evaluated according to the inclusion criteria: 8–12 years old, a primary TD diagnosis, and medication stable at least 1 month before treatment and stable for the duration of the therapy. Exclusion criteria were: a diagnosis of autism spectrum disorder or intellectual disability, receiving another behavioral treatment for tics during the study, and a problem of geographical location to assure treatment adherence. Thirteen children were originally recruited and seven children completed therapy and posttreatment measures (one retracted before therapy, four abandoned during therapy, and one completed the therapy, but did not complete the follow-up). Table 1 summarizes age, sex, medication intake, and number of days between the first and the last therapy session for each participant that completed the therapy. The mean age of the seven participants was 10.29 years (six boys, one girl). Mean age of the non-completers was 9.4 years (four boys, two girls). There was no statistical difference between completers and non-completers over all measures of tic severity in the pre-treatment assessment as shown in Table 2.

**Table 1:** **Age, sex, medication intake, and length of the therapy for each participant**.

Participant	Age	Sex	Medication intake	Days between first and last therapy session
1	11	Girl	Valerian, atomoxetine	91
2	10	Boy	–	98
3	10	Boy	–	115
4	12	Boy	–	98
5	11	Boy	Melatonin	104
6	9	Boy	Methylphenidade, risperidone	106
7	9	Boy	–	105

**Table 2:** **Tests of tic severity differences between completers and non-completers on the YGTSS and on the TSGS**.

Scale	Median score for completers (participants)	Median score for non-completers	Asymptotic Wilcoxon–Mann–Whitney Test
**YGTSS**			
Global	37.00	29.50	*Z* = −0.857, *p* = 0.39
Tic severity	23.00	19.50	*Z* = −0.714, *p* = 0.48
Deterioration	10.00	10.00	*Z* = −0.158, *p* = 0.87
**TSGS**			
Global	25.50	21.08	*Z* = −0.286, *p* = 0.78
Tic domain	13.00	10.00	*Z* = −0.644, *p* = 0.52
Social functioning domain	10.00	10.00	*Z* = 0.443, *p* = 0.66

### Assessment Measures

#### Yale Global Tic Severity Scale

The *Yale Global Tic Severity Scale* [YGTSS, Ref. (50)] is used to assess a global scale based on a tic severity subscale with five dimensions (number, frequency, intensity, complexity, and interference of tics) and an impairment subscale. Inter-rater agreement ranges from 0.52 to 0.99 and 0.85 for the global severity score. Factor loadings on the items in factor analyses revealed two separated factors, one for motor tics and overall impairment and one for phonic tics, although the two factors account only for 8% of the variance showing a low-factor validity. The YGTSS is completed by the children with the help of an independent evaluator. Scores for the YGTSS ranged from 0 to 100.

#### Tourette’s Syndrome Global Scale

The *Tourette’s Syndrome Global Scale* [TSGS, Ref. (51)] is used to assess a global scale based on a tics domain and a social functioning domain. The tics domain evaluates frequency and disruption of different subtypes of tics (motor/phonic and simple/complex). Social functioning domain included the assessment of learning, motor restlessness, and occupational problems. There is a good inter-rater agreement (0.89) for the global scale, and the criterion validity was demonstrated as a correlation between TSGS’s global scale and severity of TD symptomatology ranked by four raters ranging from 0.46 to 0.99. The TSGS highly correlates with the YGTSS for motor, phonic, and total tics (from 0.86 to 0.91), but the correlation is moderate for the global score. The TSGS is completed by the children with the help of an independent evaluator and by one of their parents. Scores for the TSGS ranged from 0 to 100.

#### Behavior Assessment System for Children – Second Edition

The *Behavior Assessment System for Children – Second Edition* [BASC-2, Ref. (52)] is a multidimensional and multimodal assessment for adaptive and clinical aspects of behavior and personality in children. Two tests were used to assess participants on secondary outcomes of the therapy, one by the parents and one by the children. The *Parent Rating Scale* (PRS) assesses nine clinical scales (hyperactivity, aggression, conduct problems, anxiety, depression, somatization, atypicality, withdrawal, and attention problems), five adaptive scale (adaptability, social skills, leadership, activities of daily living, and functional communication), three clinical composite scale (externalizing problems composite, internalizing problems composite, and behavioral symptoms index), and one adaptive composite scale (Adaptive skills composite), over 160 items. The *self-reported personality* (SRP) for children assesses 10 clinical scales (attitude to school, attitude to teachers, atypicality, locus of control, social stress, anxiety, depression, sense of inadequacy, attention problems, and hyperactivity), four adaptive scales (Relations with parents, Interpersonal relations, Self-esteem and Self-reliance), 4 clinical composite scales (school problems composite, internalizing problems composite, inattention/hyperactivity composite, and emotional symptoms index), and 1 adaptive composite scale (personal adjustment composite), over 139 items.

For both tests, scores were converted to *T*-score based on the age of the participant. Intervals of *T*-scores indicating thresholds for “normal,” “at risk,” and “clinically significant” ranges are presented in Table 3, for the clinical scales and for the adaptive scales. Internal consistency of scales and composite scales for the PRS were all above α = 0.80, and test–retest reliability were all above 0.77. For the SRP, internal consistency of scales and composite scales ranged from α = 0.71 to α = 0.96 and test–retest reliability ranged from 0.66 to 0.83. Change in time on the SRP could be attributed to low reliability. Confirmatory factor analysis for the PRS showed a comparative fit index of 0.88 and a root mean square error of approximation of 0.13, both indicating near good validity of the test. Confirmatory factor analysis results were equivalent on the SRP, with a comparative fit index of 0.90 and a root mean square error of approximation of 0.11, indicating good and near good validity of the test.

**Table 3:** **BASC-2 *T*-scores indicating thresholds scores for clinical and adaptive scales**.

Type of scales	*T*-scores				
	<30	40	50	60	>70
Clinical		Normal		At-risk	Clinical
Adaptive	Clinical	At-risk		Normal	

*The gray shade are visual indicator of the At-risk and Clinical score range for the BASC-2*.

#### Culture-Free Self-esteem Inventory

The *Culture-Free Self-Esteem Inventory – second edition form B* [CFSEI, Ref. (53)] was used to evaluate change in self-esteem in children between pre- and posttreatment as a secondary benefit of the therapy. The CFSEI form B included 30 yes or no items assessing five subscales (general, social, academic, parents, and defensiveness) extracted from form A. Correlation between the two forms was 0.86. Test–retest reliability was ranging from 0.79 to 0.92 for the total score and was ranging from 0.49 to 0.80 for subscales. Concurrent validity was obtained with the self-esteem inventory (54), ranging from 0.71 to 0.80.

### Treatment Material

The *Facotik* treatment is a manualized therapy (therapist and child manual), including a self-monitoring diary and a token economy motivational board. The therapist’s manual includes an explicit protocol for every exercise and instructions for the participants and their parents for each session of the therapy with time estimation. The child’s manual contains information on each topic of the treatment with colorful examples, activities named “challenges,” and exercises to practice between therapy sessions named “missions of the week.” A particular concern was to adapt the CoPs exercises to a child’s cognitive level of functioning. For this purpose, a narrative approach was proposed in *Facotik* where two characters named Lea and Nico accompanied the child over the treatment. To improve understanding, new elements have been added in the children adaptation of the therapy, such as concrete language, practical examples, metaphors, visual analogies, and pictures. Also, behavioral restructuring precedes cognitive restructuring, unlike the adult version. The self-monitoring diary is used for assessing frequency of tics, conducting a functional analysis (antecedents, consequences), and clinical awareness training. Each participant notes the frequency of a targeted tic for a 15-min period, once a day, in a predetermined high-risk tic onset situation. The child also estimates the intensity of the tics (low, medium, or high) and his/her principal activity at this time. The token economy motivational system works on a three-point award for each therapy session, one for participating in the challenges during the session, one for completing the self-monitoring diary every day and one for completing the weekly exercises or missions between sessions. Children could exchange nine points for a specific reward (not necessarily tangible, e.g., a specific activity), determined with their parents at the second session.

### Procedure

Participants and one of their parents completed the pre-treatment assessment with a trained specialized evaluator, including YGTSS, TSGS, BASC-2, and CFSEI. The certified evaluator was independent of the therapy process and research protocol. The evaluator completed the scoring of the YGTSS and the TSGS, after semi-structured interviews with the parents and the children separately. Afterward, each participant followed the *Facotik* therapy with one of the two trained psychotherapists: a licensed psychologist and a certified final year graduate student. The *Facotik* therapy lasted 12 to 14 sessions depending on the understanding and on the success of the steps by the child. Each 90-min session began by reviewing the content previously discussed and ended with 20 min of parental training (information on the clinical objective of the session, supportive coping strategies, and how to give positive reinforcement for home exercises to their child). Information was also given to the parents on the theoretical approach to enable them to act as a collaborator in the therapy process based on a psychoeducation method (55, 56). Psychotherapists wrote a progress report at the end of each therapy session, indicating children’s progress and difficulties.

The *Facotik* treatment is progressive and passes through progressive therapeutic steps with a “*one tic at a time*” approach. Table 4 presents a schema of the clinical objectives and the therapeutic components of each therapy session. The clinical objective distributed over 14 sessions are: awareness training, muscle discrimination, relaxation, reduced sensorimotor activation, modifying style of planning action, cognitive restructuration of anticipation and appraisals, behavioral restructuration, generalizations, and preventing relapse. The first clinical objective (awareness training) is spread over several sessions, while, from the 9th therapy session, several clinical objectives are addressed in the same sessions. Between each therapy session, the child completed the self-monitoring diary and the weekly exercises. Three participants completed treatment in 13 sessions, and four others completed therapy in 14 sessions (the total duration of the therapy was an average of 102.49 days between the first and the last session, all children skipped at least 1 week between two sessions due to sickness or scheduling constraints). At posttreatment, each participant and one of their parents completed all assessments on the YGTSS, the TSGS, the BASC-2, and the CFSEI.

**Table 4:** **Procedure, therapeutic components, and clinical objectives of each *Facotik* session**.

Clinical objectives	Session	Procedure and therapeutic components
Awareness training	1	– Introduction to the therapy; psychoeducation about TD and tics – Identifying a targeted tic (the most preoccupying or frequent) – Identifying form of tic in details (muscles involved, sequence) – Establishing a list of inconveniences to tics – Presentation of the self-monitoring diary and token economy motivational boards
	2	– Psychoeducation and presentation of the CoPs approach to managing tics – Explanation of the triple link between thoughts, feelings and global tension, and tics
	3	– Tic profiling: identifying personal high and low tic onset risk situation – Analyzing situation profiles; activities, and feelings in each of those situations? (establishing distinctions)
	4	– Cognitive and emotional analysis of high and low tic onset risk situation – Analyzing the link between thoughts (anticipations), emotions, physiological state, and actions/tics
	5	– Video recording of a high and a low tic onset risk situation (a real-life experience forms the basis for the script) – Each situation is filmed for 10 min during the session. – Viewing the scenes together with the child to analyze the differences between both situation (behavioral situational analysis)
Muscle discrimination	6	– Awareness training of muscular tension and muscular discrimination – Increasing tic muscle flexibility and gaining control over tension in the tic-affected muscles – Learning to graduate the muscle tension level through practice in slowly contracting/relaxing muscles by degree (normalize effort involved; not yet progressive muscular relaxation)
Relaxation	7	– Practicing abdominal breathing and progressive muscular relaxation to improve motor control learned with discrimination exercises and to prevent tension in everyday life
Sensory-motor activation	8	– Reducing sensory–motor activation in avoiding anticipatory vigilance to sensation and not attributing significance to sensation in high tic onset risk situations (stopping negative reinforcement process) – Identification of personal style of planning action (over-activity, over-investment)
Style of planning action	9–10	– Understanding the link between a tension-producing style of planning action and specific experienced muscle tension, and tics (reducing over-activity and over-investment) – Identifying personalized advantages and disadvantages of those styles of action; which may relate to irrational thoughts that can be addressed with cognitive restructuring – Realizing that optimal preparation is already in their person’s repertoire
Cognitive restructuring	11–13	– Modifying core beliefs about perceptions of others and related to style of action planning – Activities at high-risk tic onset are evaluated for the presence of beliefs and judgments about the activity likely to impede optimal planning – Addressing perfectionist thinking and irrational thoughts on how to behave
Behavioral restructuring	11–13	– Modifying preparation for a situation (e.g., prevention by relaxation) – Eliminating tension-producing strategies to inhibit or disguise the tic (e.g., holding in the tic) – Highlighting existing abilities rather than learning a new response
Global restructuring	11–13	– Cognitive, sensorimotor, emotional, and behavioral components of this planning can be addressed at the same time during cognitive–behavioral modification – Cognitive and behavioral restructuring are two steps integrated during the last session of global restructuring – Generalizing practice to different situations
Generalization	14	– Applying strategies to other high-risk situations or to unforeseen situations – Applying strategies to other tics or behavior
Relapse prevention	14	– Keep practicing, refresh knowledge, and maintain gains – Anticipate situations that may trigger relapse of tics and change other aspects of life style – Feedback and therapy conclusion

### Ethics

This study was approved by the local ethic review board of the *Institut universitaire en santé mentale de Montréal* in accordance with the ethical standards of the *Canadian Tri-Council Policy Statement of Ethical Conduct for Research Involving Humans*. The parents of the participants (or the legal guardian) gave their signed consent for the participation of their child to the study (assessments and therapy), and the child himself gave his or her approval.

### Data Analyses

Two analysis procedures were planned. For statistical analyses, one-sided exact Wilcoxon signed-rank test was conducted due to the small sample on the children and parents’ assessments to evaluate global symptoms decrease after treatment, as measured by the YGTSS global scale and the TSGS global scale. Additional one-sided exact Wilcoxon signed-rank tests were conducted with the tic severity subscale and the impairment subscale of the YGTSS and with the tics domain and the social functioning domain of the TSGS, using a Pratt correction in the case of tied ranks. Person’s correlations were computed between parents and children for pre-treatment scores, posttreatment scores, and difference scores on all scales and subscales. Difference scores were computed as pre-treatment score minus posttreatment score for each parents and children. All analyses were calculated with *n* = 7 based on a complete dataset. All statistical analyses were computed using R statistical software (57) and the coin package (58). For clinical results, changes of at least 1 SD on the BASC-2 subscales and on the CFSEI were reported.

## Results

### Statistical Results

**Figure 1:**
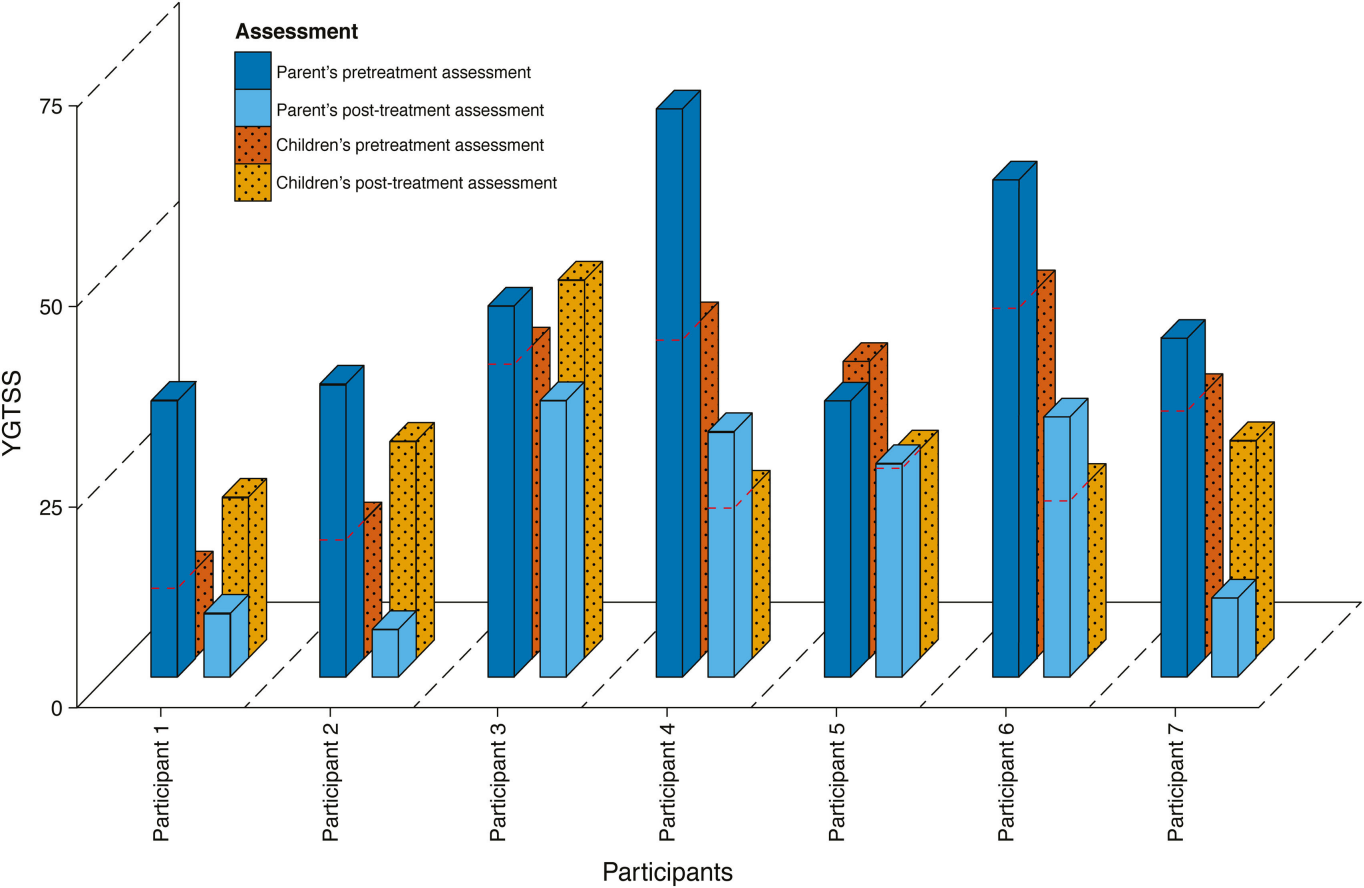
**Results on the YGTSS for parents and children in pre- and posttreatment**.

Results of the parents’ assessments on the YGTSS global scale showed a general and significant symptom decrease at posttreatment (from Mdn = 43.00 to Mdn = 27.00, *Z* = −2.37, *p* = 0.008, *r* = −0.63). This decrease was not perceived by the children themselves, as they estimated no significant symptoms decrease (from Mdn = 37.00 to Mdn = 26.00, *Z* = −0.85, *p* = 0.234). Figure 1 shows the global scale on the YGTSS for pre- and posttreatment as assessed by the children and their parent. Four participants showed a decrease on the YGTSS global scale for both child and parent, while the other three reported discrepant results. Correlations between parents and children showed good agreement for pre-treatment scores (*r* = 0.70, *p* = 0.005), but poor agreement for posttreatment scores (*r* = 0.34, *p* = 0.234) and for difference scores (*r* = 0.30, *p* = 0.300).

Analysis of the YGTSS subscales showed a significant decrease in both the tic severity subscale (Mdn = 27.00 to Mdn = 15.00, *Z* = −2.37, *p* = 0.008, *r* = −0.63) and the impairment subscale (Mdn = 20.00 to Mdn = 10.00, *Z* = −2.19, *p* = 0.031, *r* = −0.69), as observed by parents. Children reported a significant decrease on the tic severity subscale (Mdn = 23.00 to Mdn = 16.00, *Z* = −2.29, *p* = 0.016, *r* = −0.66), but not on the impairment subscale (Mdn = 10.00 to Mdn = 10.00, *Z* = 0.81, *p* = 0.813). Correlations between parents and children on the tic severity subscale were moderate for pre-treatment scores (*r* = 0.60, *p* = 0.023), negative for posttreatment scores (*r* = -0.35, *p* = 0.220), and poor for difference scores (*r* = 0.29, *p* = 0.315). Correlations between parents and children on the impairment subscale were poor for pre-treatment scores (*r* = 0.61, *p* = 0.021), good for posttreatment scores (*r* = 0.75, *p* = 0.002), and moderate for difference scores (*r* = 0.50, *p* = 0.069).

**Figure 2:**
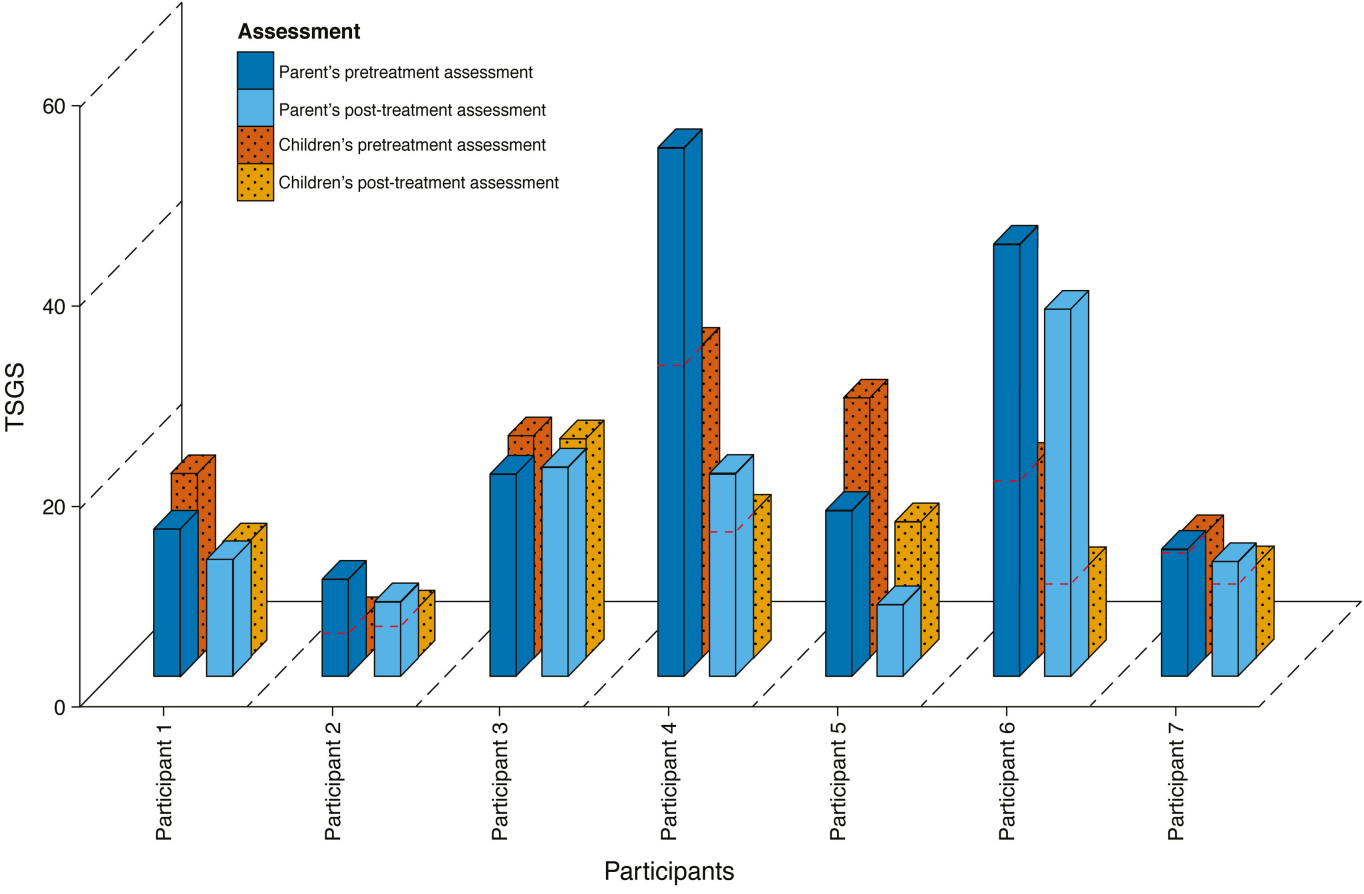
**Results on the TSGS for parents and children in pre- and posttreatment**.

In contrast to YGTSS, results on the TSGS global scale showed a significant symptom decrease after treatment, as assessed by children (from Mdn = 25.50 to Mdn = 11.67, *Z* = −2.37, *p* = 0.008, *r* = −0.59), and by the parents (from Mdn = 16.83 to Mdn = 12.00, *Z* = −2.20, *p* = 0.016, *r* = −0.59). Figure 2 shows scores on the TSGS global scale for pre- and posttreatment as assessed by children and parents for each participant. Five participants showed a decrease in tic symptoms on the TSGS global scale, while a further two reported discrepant results. Correlations between parents and children showed good agreement for pre-treatment scores (*r* = 0.74, *p* = 0.002), poor agreement for posttreatment scores (*r* = 0.19, *p* = 0.515), and moderate agreement for difference scores (*r* = 0.47, *p* = 0.090).

Analysis of the TSGS domains as reported by parents showed a significant decrease in the tics domain (Mdn = 13.00 to Mdn = 4.00, *Z* = −2.37, *p* = 0.008, *r* = −0.63), but not on the social functioning domain (Mdn = 10.00 to Mdn = 10.00, *Z* = −0.71, *p* = 0.750). Children reported a significant decrease on tics domain (Mdn = 13.00 to Mdn = 6.00, *Z* = −2.37, *p* = 0.008, *r* = −0.63), but not on the social functioning domain (Mdn = 10.00 to Mdn = 6.67, *Z* = 0.78, *p* = 0.281). Correlations between parents and children on the tics domain were good for pre-treatment scores (*r* = 0.75, *p* = 0.002), moderate for posttreatment scores (*r* = −0.41, *p* = 0.145), and good for difference scores (*r* = 0.77, *p* = 0.001). Correlations between parents and children on the social functioning domain were moderate for pre-treatment scores (*r* = 0.55, *p* = 0.042), poor for posttreatment scores (*r* = 0.20, *p* = 0.493), and negative for difference scores (*r* = −0.11, *p* = 0.708).

### Clinical Results

The BASC-2 and the CFSEI were used to detect if the *Facotik* therapy brought secondary benefits to develop adaptive and clinical aspects of behaviors and self-esteem. Table 5 showed clinical changes of at least 1 SD on the BASC-2 SRP and on the BASC-2 *Parent rating scale* (PRS). There were no globally significant changes over participants even if there were some changes at the individual level. For all participants and all clinical subscales together, parents reported improvements in 13 subscales and decreases in 6 subscales, while the children report 9 improvements and 9 decreases. An overall decrease is observed for participant 1 (as noted by the parent) and participant 7 (as noted by the child). Participant 6 is the only one to present only slight increases observed by the parent (atypicality) and the child (anxiety, attention problems). However, children scoring shows that the attitude toward school, teachers, and the school problems composite increased slightly for three participants. All other participants showed decreases and increases in some subscales without general trend. For the adaptive scales, improvements are observed by parents in five subscales for the seven participants (adaptability for two participants, leadership, functional communication, and adaptive skills). Children have noted improvements in three subscales (self-esteem for two participants and self-reliance) and one decrease (interpersonal relations). All these results are not significant, but showed clinical changes of at least 1 SD.

**Table 5:** **Clinical change between pre- and posttreatment on the BASC-2^a^**.

		Participant 1		Participant 2		Participant 3		Participant 4		Participant 5		Participant 6		Participant 7	
		Pre	Post	Pre	Post	Pre	Post	Pre	Post	Pre	Post	Pre	Post	Pre	Post
**(A) Data from the Parent Rating Scale (PRS)**															
Clinical scales	Conduct problems	65	51	–	–	–	–	–	–	–	–	–	–	–	–
	Externalizing problems	62	52	–	–	–	–	–	–	–	–	–	–	–	–
	Anxiety	72	49	–	–	–	–	57	72	–	–	–	–	–	–
	Depression	68	52	11	49	–	–	67	54	–	–	–	–	–	–
	Somatization	–	–	67	47	53	36	–	–	44	56	–	–	44	70
	Internalizing problems	65	46	–	–	53	40	–	–	–	–	–	–	–	–
	Atypicality	65	52	–	–	–	–	44	54	–	–	49	65	–	–
	Withdrawal	69	56	–	–	–	–	65	54	–	–	–	–	–	–
	Behavioral symptoms index	68	56	–	–	–	–	–	–	–	–	–	–	–	–
Adaptive scales	Adaptability	–	–	–	–	32	53	–	–	–	–	16	28	–	–
	Leadership	–	–	–	–	38	51	–	–	–	–	–	–	–	–
	Functional communication	–	–	30	55	–	–	–	–	–	–	–	–	–	–
	Adaptive skills	–	–	–	–	40	53	–	–	–	–	–	–	–	–
**(B) Data from the self-reported personality (SRP)**															
Clinical scales	Attitude to school	–	–	–	–	–	–	–	–	45	61	–	–	–	–
	Attitude to teachers	49	71	–	–	–	–	36	49	–	–	–	–	–	–
	School problems composite	52	68	–	–	–	–	–	–	42	52	–	–	–	–
	Atypicality	–	–	–	–	–	–	–	–	–	–	–	–	59	45
	Locus of control	–	–	–	–	53	42	–	–	51	37	–	–	58	46
	Social stress	13	48	–	–	50	64	–	–	–	–	–	–	52	37
	Anxiety	–	–	–	–	–	–	–	–	–	–	39	51	62	47
	Depression	–	–	–	–	–	–	–	–	–	–	–	–	61	45
	Internalizing problems composite	–	–	–	–	–	–	–	–	–	–	–	–	57	42
	Attention problems	–	–	–	–	–	–	–	–	–	–	40	51	–	–
	Emotional symptoms index	–	–	–	–	–	–	–	–	–	–	–	–	54	40
Adaptive scales	Interpersonal relations	–	–	–	–	50	38	–	–	–	–	–	–	–	–
	Self-esteem	–	–	–	–	41	58	–	–	–	–	–	–	47	58
	Self-reliance	–	–	–	–	47	59	–	–	–	–	–	–	–	–

^a^(A) data from the Parent Rating Scale (PRS); (B) data from the self-reported personality (SRP). Only scores that changed for at least 1 SD (10 *T*-score) are shown. Clinical scales: scores ≥ 60 are “at-risk”; scores ≥ 70 are “clinically significant.” Adaptive scales: scores ≤ 40 are “at-risk”; scores ≤ 30 are “clinically significant.”

One child showed improvement on self-esteem as measured by the CFSEI, with an increase on the total score of 2 SD, the global subtest of 1.5 SD, the parent subtest of 1 SD and the academic subtest of 2.7 SD. All other participants maintained medium to high levels of self-esteem from pre- to posttreatment. Table 6 shows data for all participants on the CFSEI.

**Table 6:** ***T*-score on the CFSEI for each participant in pre- and posttreatment on each scale**.

	Total score		Global subtest		Parent subtest		Academic subtest		Social subtest	
	Pre	Post	Pre	Post	Pre	Post	Pre	Post	Pre	Post
Part 1	63	60	65	65	50	50	63	63	55	46
Part 2	63	65	60	65	60	60	63	63	55	55
Part 3	55	52	55	55	60	60	54	54	46	38
Part 4	63	63	60	60	60	60	63	63	55	46
Part 5	65	68	65	65	60	60	63	63	55	55
Part 6	60	63	60	60	60	60	63	63	46	46
Part 7	45	65^a^	50	65^a^	50	60^a^	36	63^a^	46	46

*^a^Change in T-score of at least 1 SD*.

## Discussion

### Principal Results

The purpose of the current study was to evaluate the efficiency of the *Facotik* treatment to decrease the severity of tics in children aged 8–12 years old. Secondary benefits to improve adaptive and clinical aspects of behaviors and self-esteem were also anticipated.

The overall results showed a significant decrease in tics as assessed by the parents of children with TD. The results as assessed by children were discrepant; tics decreased significantly for all children as measured with the TSGS and four participants on seven reported a non-significant decrease on the YGTSS. However, children and parents, all reported a significant decrease in tic severity when the subscales of the two questionnaires were analyzed. What is interesting is that, even considering this change in tics, children and parents generally perceive no changes in the impairment subscale. This could be explained by the presence of comorbidity symptoms, which was not controlled in this study or by the subjective experience of the impairment. The correlations between the child/parents’ rating showed a good agreement regarding the tic severity in pre-treatment, but not in posttreatment, neither for difference scores (pre-minus posttreatment), suggesting a disagreement about the perception of change. There are two possible explanations for the preceding results. First, the difference between the child/parents’ rating on the YGTSS and the TSGS may highlight the sensitivity of the TSGS, which is multidimensional and is rated on a scale rather than in categories as in the YGTSS. Second, the tic decrease might not always be detected by the children themselves and discrepancies between the child/parents’ rating may be explained by one of the therapy components termed “awareness training” (59). Children are more aware of their tics after the therapy and they can detect and report them more accurately than at pre-treatment, while the parents noticed a decrease of tics because they were already conscious of the tics. The self-monitoring diaries are a key component of the tic awareness training (60). The focus on a single tic may help children to acknowledge the difference between a situation with high risk of tic onset versus low-risk situations. Some situations may be perceived as a high risk in the first place, but may become low risk following the self-monitoring diary. Thus, the mixed results may be more of an indication of the therapy process than an absence of progress in tic reduction.

Adaptive and clinical aspects of behaviors in children, as measured by the BASC-2, showed no significant changes, but improvements and clinical changes were reported individually, suggesting a regular fluctuation over time. There are further improvements to clinical subscales than deterioration as reported by children and parents. As an example, internalizing problems showed punctual improvement. Improvements have also been noticed in general for the adaptive scales. This highlights positive results although there are no significant differences. All the participants maintained or reached medium to high levels of self-esteem from pre- to posttreatment. However, attitude toward school or teacher appear to have increased for three participants after therapy. This could be explained by the fact that the therapy ended concurrently with or after the end of the school year, and posttreatment assessment took place (particularly for participants 4 and 5) just before the return to school period (in August).

In terms of experiential factors, all children benefited from the therapy, and no adverse effects were reported by the participants or their parents. The participants reported to the therapists that theoretical concepts and exercises were presented in a clear and colorful way, which made them comprehensive for all, even for participant 4 who had language issues; some activities took more time, but without causing a significant delay. Some children had a little trouble to identify their irrational thoughts during high-risk tic onset situations, and all participants reported that completing their self-monitoring diary and relaxation exercises were most helpful to them. According to the therapists, the set of strategies formed a coherent whole, and children were open-minded to the complementary elements of the therapy; they were particularly interested when the style of action planning was addressed.

### Limitations

The limitations of the present study are those inherent in a consecutive case series without baseline or control group and a limited number of participants. The attrition rate was around 40%, but there were no clinical or demographical differences between participants and those who abandoned. Personal motivation and difficulty scheduling therapy sessions appear to account for attrition. This protocol had a confounding variable, considering that the posttreatment was concomitant with the preparation of a new school year. This situation could have an impact on tics and on clinical aspects of behaviors. A 6- and 12-month follow-up assessment is planned. The participants were prescribed a variety of medications, and comorbidities were not controlled. Nonetheless, the statistical and clinical significance of the tic reduction indicates potential efficacy of the *Facotik* treatment.

### Future Research

The main strength of the current study is the demonstration of the effect of the *Facotik* treatment for the decrease of tic severity in children as a first step of the validation procedure. These findings, with a manualized treatment and a structured protocol, highlight the clinical importance of working on the cognitive and central processes underlying tics in children as in adults (40). CoPs treatment in adults has been shown to produce neurocognitive changes in style of action and concomitant cerebral functioning (61, 62). Such physiological changes (activation of the pre-motor and motor cortex) related to the intervention remain to be validated in children with tics (61, 62). In conclusion, this study has important implications for the conceptualization of interventions in TD; namely to know if tics are the necessary and sufficient target for effective interventions or if the processes underlying tics should also be addressed to obtain greater symptom reduction and wider behavioral impact. Future research will include a randomized clinical trial design where the efficacy of *Facotik* treatment as well as CoPs treatment in adults is compared to CBIT (2015–2020). Follow-up data and the effect of the therapy on quality of life for all the participants of the present study are still pending. Finally, the *Facotik* therapy manual will be published as a workbook for therapists and specialized training will be offered to clinicians to facilitate knowledge transfer.

## Author Contributions

JL created a new therapy for children with tics (Facotik). She oversaw the project (e.g., method, ethics, supervision) and coordinated the writing of the article with a focus on the results analysis and the discussion. KO is the author of the conceptual model that led to the new therapy for children presented in this article. He is the principal researcher on the grant that supported this study. He revised the text and helped with the data analysis. GJ-N contributed to the writing of the manuscript, especially the introduction and the review of the literature. PV contributed to the writing and the text formatting and was in charge of the statistical analysis. ML revised the manuscript and was on the funding grant that supported this study. His research focus is on psychophysiological data and on event-related potentials [see other article in the same topic: Morand-Beaulieu et al. (62)].

## Conflict of Interest Statement

The authors declare that the research was conducted in the absence of any commercial or financial relationships that could be construed as a potential conflict of interest. The reviewer EJ and handling Editor declared their shared affiliation, and the handling Editor states that the process nevertheless met the standards of a fair and objective review.
